# Altered expression of major immune regulatory molecules in peripheral blood immune cells associated with breast cancer

**DOI:** 10.1007/s12282-016-0682-7

**Published:** 2016-03-04

**Authors:** Kosuke Kawaguchi, Eiji Suzuki, Ayane Yamaguchi, Michio Yamamoto, Satoshi Morita, Masakazu Toi

**Affiliations:** 1 Department of Breast Surgery, Graduate School of Medicine, Kyoto University, 54 Shogoin-kawaharacho, Sakyo-ku, Kyoto, 606-8507 Japan; 2 Department of Biomedical Statistics and Bioinformatics, Kyoto University, Kyoto, Japan

**Keywords:** Breast cancer, PD-1, CD80, PD-L1, CD40 and PBMCs

## Abstract

**Background:**

The purpose of this study was to clarify the alterations of major immune regulators in peripheral blood mononuclear cells (PBMCs) of cancer patients and to analyze the association with the disease progression in breast cancer patients.

**Methods:**

The study included 6 healthy volunteers (HVs), 12 primary breast cancer (PBC) patients, and 30 metastatic breast cancer (MBC) patients. The expression of immune regulators such as, *CCR6*, *CD4*, *CD8*, *CD14*, *CD40*, *CD56*, *CD80*, *CTLA4*, *CXCR4*, *FOXP3*, *IDO*-*1*, *IDO*-*2*, *NKG2D*, *NRP*-*1*, *PD*-*1*, and *PD*-*L1* mRNA in PBMCs was measured by quantitative RT-PCR. Analysis of variance with contrasts was performed to find expression patterns of the three groups (HVs, PBC, MBC).

**Results:**

We clarified the alterations of mRNA of major immune regulators *PD*-*L1, FOXP3, CD80, CD40,* and *CD14* in PBMCs of cancer patients and the association of these alternations with disease progression. Furthermore, *PD*-*L1* expression was correlated with serum interferon-γ production.

**Conclusion:**

Our data suggested that mRNA expressions of *PD*-*L1, FOXP3, CD80, CD40* and *CD14* in PBMCs are affected by disease progression. Understanding the roles of these various interactions will be of importance to future studies aiming to uncover biomarkers for predicting response to immune therapy.

## Introduction

Recent clinical data have emphatically shown the capacity of our immune systems to eradicate even advanced cancers. Comparably high response rates were reported in initial clinical trials evaluating inhibitors of the immune checkpoint, such as anti-PD-L1, anti-PD-1, and anti-CTLA4, in various cancers [1–6]. However, the objective response rate to inhibitors of the immune checkpoint was 30–40 % and it was unclear which biomarkers could be used to predict the clinical response to immune checkpoint inhibitors. Thus, biomarker analysis of immune checkpoint inhibitors is a research priority.

Recent biomarker analysis showed mismatch repair-deficient tumors and tumor-specific neoantigen load were highly responsive to checkpoint blockade with anti-PD-1 [7]. Other studies suggested that hypermutated tumors might harbor additional tumor-specific neoantigens and increased amounts of tumor-infiltrating lymphocytes (TILs) [8–10]. Thus, PD-L1 expression by TILs rather than tumor cells is more predictive of the response to blockade of the PD-1 pathway [11, 12]. Although these biomarkers are meaningful, they have problems of assay complexity with respect to clinical usage, cost, repeatability, and heterogeneity.

The purpose of this study was to confirm that peripheral blood immune cells, and not immune cells, in tumor tissue can be used to evaluate immune checkpoint-related gene expression in terms of the correlation of expression level with the clinical status of breast cancer. We prospectively validated PBMCs in healthy volunteers (HVs), primary breast cancer (PBC) patients, and metastatic breast cancer (MBC) patients. At the molecular level, the mRNA expression levels of 16 immune genes were measured in PBMCs, including putative immunosuppressive factors (*IDO*-*1*, *PD*-*1*, *PD*-*L1*, *CTLA4*, and *FOXP3*). The rationale for marker selection was to include T cell markers, chemokines, and immune checkpoint markers that are currently under evaluation as therapeutic targets.

## Materials and methods

### Study design and outcomes

**Fig. 1:**
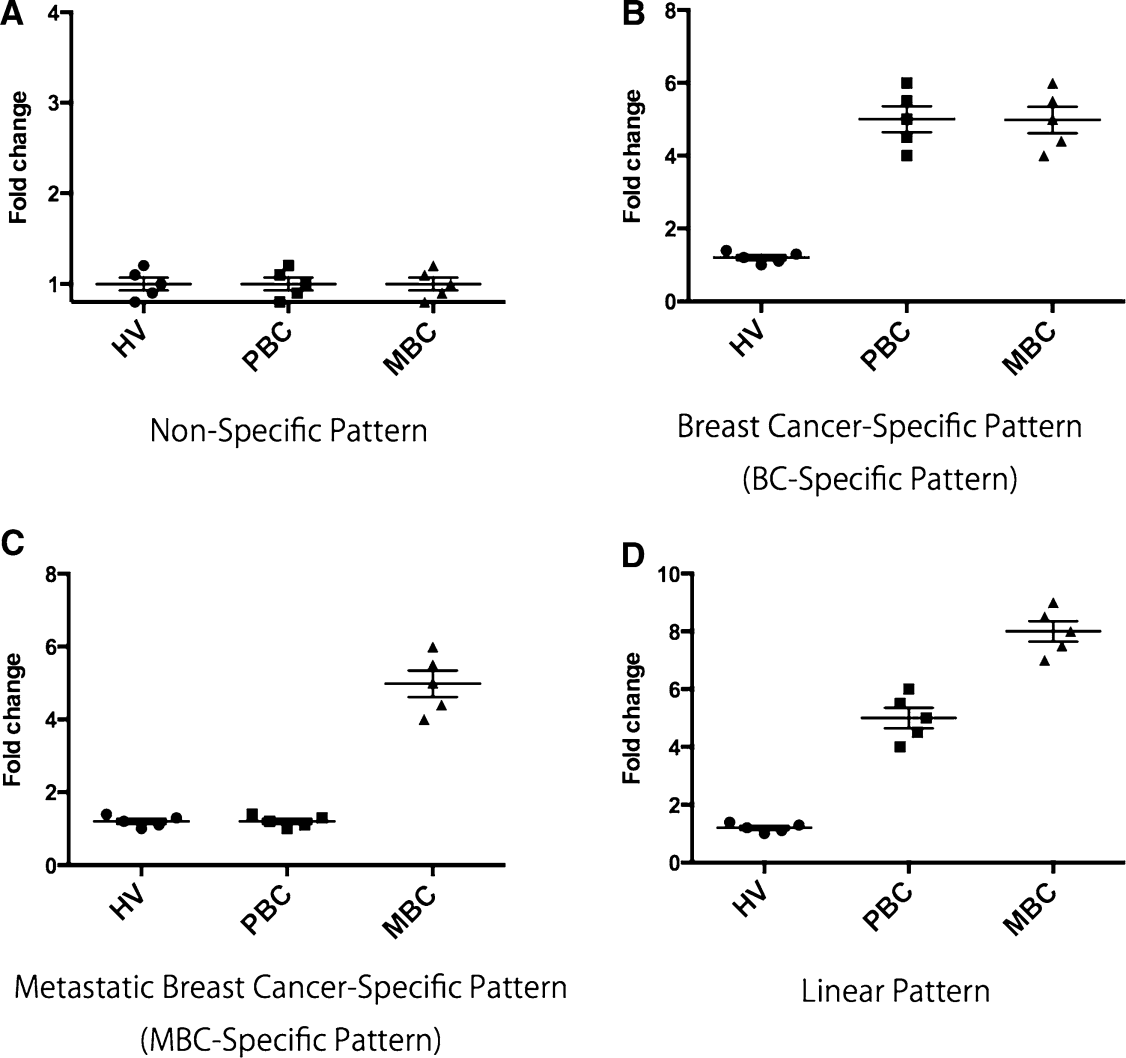
Representative examples of *box plots* of four expression patterns. **a** Non-specific pattern. **b** Breast cancer-specific pattern. **c** Metastatic breast cancer-specific pattern. **d** Linear pattern

We screened 16 genes (*CD80*, *CTLA4*, *IDO*-*1*, *IDO*-*2*, *PD*-*1*, *PD*-*L1*, *CD56*, *FOXP3*, *NKG2D*, *NRP*-*1*, *CD4*, *CD8*, *CD40*, *CCR6*, *CD14*, and *CXCR4*) in PBMCs by quantitative real time-PCR (qRT-PCR). Expression patterns were defined as non-specific, breast cancer (BC)-specific, MBC-specific, and linear by ANOVA (details of statistical analysis are described below). Representative box plots of non-specific, BC-specific, MBC-specific, and linear expression patterns are presented in Fig. 1.

### Human tissue samples

All samples from HVs and BC patients were collected in the Department of Breast Surgery, Kyoto University Hospital. In PBC patients, PBMCs and serum were collected at the diagnosis. In MBC patients, PBMCs and serum were collected at the diagnosis of primary metastasis or during therapy for metastasis. Written informed consent was given by all participants prior to collection. All study protocols were approved by the Ethics Committee for Clinical Research, Kyoto University Hospital (authorization number G424) and were in keeping with the provisions of the Declaration of Helsinki in 1995.

### PBMC isolation and RNA extraction

PBMCs were prepared using BD Vacutainer CPT Cell Preparation Tubes (BD, Franklin Lakes, NJ, USA). The blood processing reported below was according to the manufacturer’s instructions. The tubes were centrifuged at room temperature for 20 min in a horizontal rotor at 1800 relative centrifugal force (RCF) within 1 h of blood collection. The plasma layer and the cells from both CPT tubes were transferred to one conical centrifuge tube. Phosphate-buffered saline was added to a final volume of 2 mL, the tubes were capped, and the cells were mixed by inversion. Subsequently, the tubes were centrifuged for 10 min at 4 °C and 1500–1800 RCF. The supernatant was aspirated and 1 mL TRIzol^®^ reagent (Invitrogen, Carlsbad, CA, USA) was added to isolate total RNA according to the manufacturer’s instructions. Total RNA was frozen immediately in liquid nitrogen and stored at −80 °C. The quality of total RNA was determined using microfluidic electrophoresis (Bioanalyzer; Agilent Technologies, Palo Alto, CA, USA).

### qRT-PCR

qRT-PCR was performed with TaqMan Fast Virus 1-step master mix (Life Technologies Carlsbad, CA, USA) and TaqMan Gene Expression probes for *CD80* (Assay ID: Hs01045163_m1), *CTLA4* (Assay ID: Hs00175480_m1), *IDO*-*1* (Assay ID: Hs00984148_m1), *IDO*-*2* (Assay ID: Hs01589373_m1), *PD*-*1* (Assay ID: Hs01550088_m1), *PD*-*L1* (Assay ID: Hs01125301_m1), *CD56* (Assay ID: Hs00941830_m1), *FOXP3* (Assay ID: Hs01085834_m1), *NKG2D* (Assay ID: Hs00183683_m1), *NRP*-*1* (Assay ID: Hs00826128_m1), *CD4* (Assay ID: Hs01058407_m1), *CD8* (Assay ID: Hs00233520_m1), *CD40* (Assay ID: Hs01002913_g1), *CCR6* (Assay ID: Hs01890706_s1), *CD14* (Assay ID: Hs02621496_s1), and *CXCR4* (Assay ID: Hs00607978_s1) (Life Technologies).

### Cytokine measurement

The cytokines in serum—interferon (IFN)-γ, transforming growth factor (TGF)-β1, TGF-β2, and TGF-β3—were measured using a Bio-Plex multiplex assay system (Bio-Rad, Hercules, CA, USA) according to the manufacturer’s instructions.

### Statistical analysis

All mRNA expression levels were normalized by their mean expression levels in HVs. ANOVA with contrasts was performed to find expression patterns of the three groups (HV, PBC, MBC). The coefficients of the contrasts for group means were as follows: (−1.0, 0.5, 0.5) for BC-specific type, (−0.5, −0.5, 1.0) for MBC-specific type, and (−1.0, 0.0, 1.0) for linear type. A gene that was non-significant for all contrasts was classified as non-specific type. Pearson correlation coefficients between expressions in PBMCs (PD-L1 and FOXP3) and cytokine levels in serum in MBC patients were calculated. Subgroup analysis in MBC was performed by Student’s *t* test. Each hypothesis was tested at the 5 % significance level. ANOVA was performed by using SAS version 9.3 software. Pearson correlation coefficient and Student’s *t* test were performed using STATA version 13.0.

## Results

### Patients’ characteristics

The characteristics of the patients are presented in Table 1. Peripheral blood samples were taken from 42 BC patients and 6 HVs. Twelve patients were PBC (28.6 %) and 30 patients were MBC (71.4 %). The main phenotype was luminal type, 58.3 % in PBC and 73.3 % in MBC. In MBC, the main metastatic type was visceral metastasis (76.7 %). Nine patients (30.0 %) had one metastatic site, seven patients (23.3 %) had two metastatic sites, twelve patients (40.0 %) had three metastatic sites and two patients (6.7 %) had four metastatic sites. About a half of patients (46.7 %) was received endocrine therapy and all HER2 positive patients were received anti-HER2 therapy.

**Table 1:** Patients’ characteristics

Characteristic	No.	%	Characteristic	No.	%
All	42	100			
Primary breast cancer (PBC)	12	28.6			
Metastatic breast cancer (MBC)	30	71.4			
PBC			MBC		
PBC ALL	12	100	MBC ALL	30	100
Age (median, range)	54.5	35–78	Age (median, range)	61	43–80
Stage			Type of metastasis	No.	%
DCIS	3	25.0	Visceral	23	76.7
I	1	8.3	Non-visceral	7	23.3
II	6	50.0	Number of metastatic sites	No.	%
III	4	33.3	1	9	30.0
Phenotype			2	7	23.3
Luminal	7	58.3	3	12	40.0
HER2	3	25.0	4	2	6.7
TNBC	1	8.3	Therapeutic status	No.	%
			Endocrine therapy	14	46.7
			Anthracycline	1	3.3
			Taxan	9	30.0
			5FU	4	13.3
			Ant-HER2 therapy	7	23.3
			Phenotype		
			Luminal	21	70.0
			HER2	7	23.3
			Triple negative	2	6.7

### mRNA expression levels in PBMCs

**Fig. 2:**
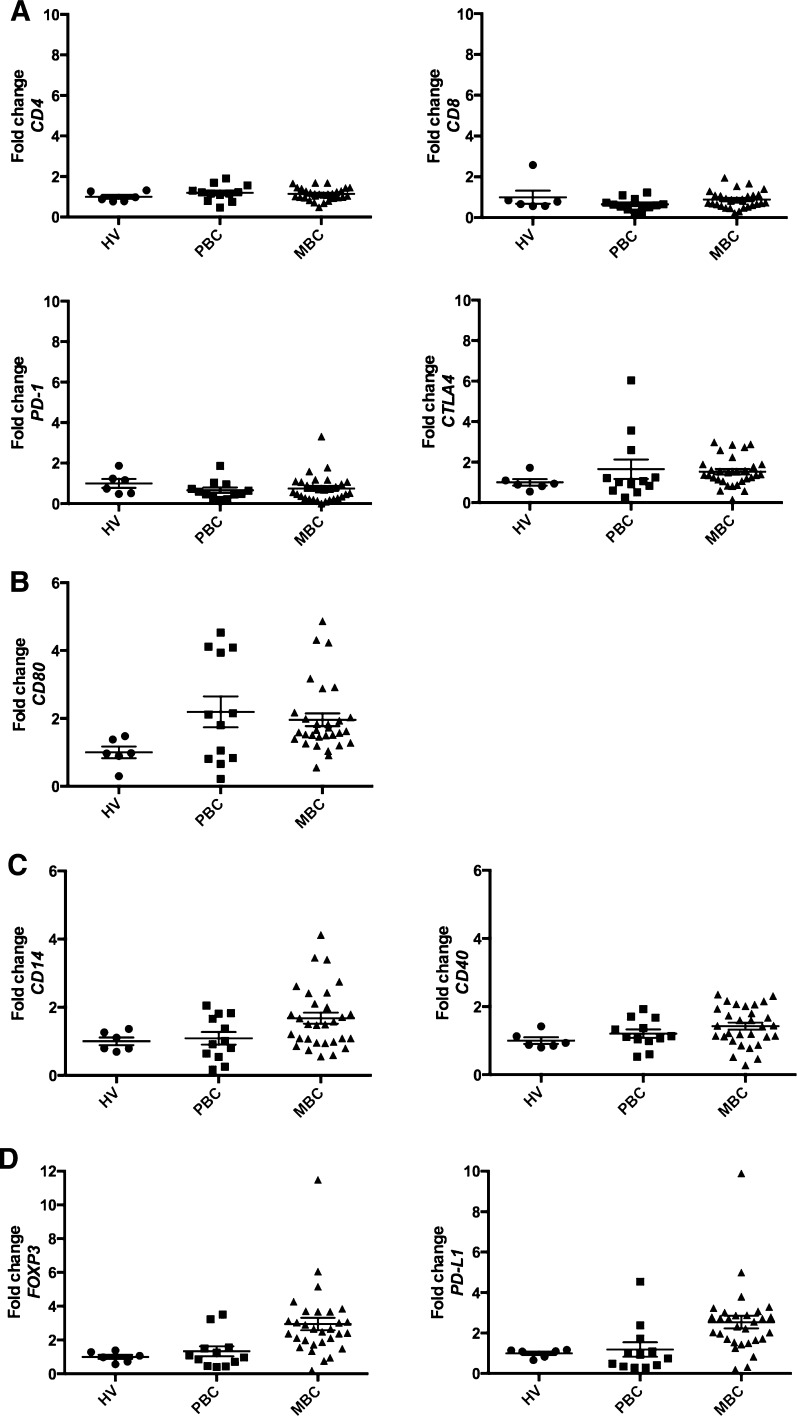
Represent figures of mRNA expression levels in PBMCs. These data show the mRNA expression levels in PBMCs by quantitative real-time-PCR. All data are normalized by the mRNA expression levels of healthy volunteers (mean 1.00). **a** Non-specific pattern: *CD4*, *CD8*, *PD*-*1*, and *CTLA4.* **b** Breast cancer-specific pattern: *CD80.* **c** Metastatic breast cancer-specific pattern: *CD14* and *CD40.* **d** Linear pattern: *PD*-*L1* and *FOXP3. All bars* show mean ± SEM

Tables 2 shows the mRNA expression levels in PBMCs. All data were normalized by the mRNA expression levels of HVs. By statistical analysis, *CD80* (*p* = 0.039) was the only gene defined as BC-specific. *CD14* (*p* = 0.046) and *CD40* (*p* = 0.013) were defined as MBC-specific, while *PD*-*L1* (*p* = 0.027) and *FOXP3* (*p* = 0.015) were defined as linear. *PD*-*L1* (*p* = 0.003) and *FOXP3* (*p* = 0.002) were also defined as MBC-specific. The mRNA levels of *PD*-*L1* and *FOXP3* in MBC were 2.54- and 2.94-fold compared with HVs, respectively. Representative box plot figures of non-specific, BC-specific, MBC-specific, and linear expression patterns are shown in Fig. 2a, b, c, d.

**Table 2:** mRNA expression levels in PBMCs

*Gene* name	Fold change (mean ± SD)			ANOVA (*p* value)			Type
	HV	PBC	MBC	BC specific	MBC specific	Linear	
***CD80***	1.000 ± 0.171	2.192 ± 0.454	1.960 ± 0.187	**0.039**	0.308	0.066	**BC-specifc**
*CTLA4*	1.000 ± 0.162	1.651 ± 0.480	1.532 ± 0.132	0.195	0.515	0.248	Non-specific
*IDO1*	1.000 ± 0.140	1.935 ± 0.519	1.297 ± 0.202	0.277	0.665	0.602	Non-specific
*IDO2*	1.000 ± 0.449	0.777 ± 0.273	0.669 ± 0.142	0.472	0.416	0.396	Non-specific
*PD1*	1.000 ± 0.217	0.659 ± 0.134	0.749 ± 0.121	0.275	0.670	0.359	Non-specific
***PDL1***	1.000 ± 0.085	1.188 ± 0.355	2.540 ± 0.313	0.201	**0.003**	**0.027**	**Linear**
*CD56*	1.000 ± 0.214	1.481 ± 0.427	1.757 ± 0.253	0.303	0.221	0.214	Non-specific
***FOXP3***	1.000 ± 0.129	1.330 ± 0.298	2.944 ± 0.373	0.142	**0.002**	**0.015**	**Linear**
*NKG2D*	1.000 ± 0.131	0.768 ± 0.175	1.323 ± 0.147	0.886	0.055	0.320	Non-specific
*NRP1*	1.000 ± 0.287	2.185 ± 0.601	1.447 ± 0.305	0.286	0.784	0.561	Non-specific
*CD4*	1.000 ± 0.098	1.196 ± 0.116	1.143 ± 0.054	0.236	0.652	0.322	Non-specific
*CD8*	1.000 ± 0.319	0.655 ± 0.087	0.886 ± 0.073	0.245	0.668	0.567	Non-specific
***CD40***	1.000 ± 0.096	1.205 ± 0.122	1.424 ± 0.103	0.167	**0.046**	0.068	**MBC-specific**
*CCR6*	1.000 ± 0.648	1.075 ± 0.616	0.744 ± 0.116	0.874	0.464	0.658	Non-specific
***CD14***	1.000 ± 0.115	1.089 ± 0.186	1.680 ± 0.165	0.282	**0.013**	0.063	**MBC-specific**
*CXCR4*	1.000 ± 0.255	1.272 ± 0.287	1.173 ± 0.168	0.618	0.720	0.621	Non-specific

All mRNA expression levels were normalized by their mean expression levels in HVs. ANOVA with contrasts was performed to find expression patterns of the three groups (HV, PBC, MBC). The coefficients of the contrasts for group means were as follows: (−1.0, 0.5, 0.5) for BC-specific type, (−0.5, −0.5, 1.0) for MBC-specific type, and (−1.0, 0.0, 1.0) for linear type. Each hypothesis was tested at the 5 % significance level. Bold font represents significant genes by ANOVA

### Subgroup analysis of *CD80*, *PD*-*L1*, *FOXP3, CD40* and *CD14* mRNA expression in MBC patients

The condition of host immune cells may be affected by various factors. Thus, we checked how *CD80*, *PD*-*L1*, *FOXP3, CD40* and *CD14* expression was affected by age, number of metastatic sites and therapeutic status in MBC patients. Table 3 shows results of subgroup analysis in MBC patients. There were no factors, which affect *CD80*, *PD*-*L1*, *FOXP3* and *CD14* expression in PBMCs in MBC patients. In CD40 expression, patients who received anti-HER2 therapy was significantly decreased compared with patients who received no prior therapy (mean mRNA levels 1.08 vs. 1.76, *p* = 0.03).

**Table 3:** Subgroup analysis of *CD80*, *PD*-*L1*, *FOXP3, CD40 and CD14* mRNA expression in MBC patients

Effect	No.	CD80			PDL1			FOXP3			CD40			CD14		
		Mean	SD	*p* value	Mean	SD	*p* value	Mean	SD	*p* value	Mean	SD	*p* value	Mean	SD	*p* value
Age																
≤60	13	1.79	1.07		3.12	2.35		3.14	2.64		1.45	0.58		1.50	0.98	
>60	17	2.09	1.00	0.44	2.10	0.84	0.11	2.79	1.50	0.66	1.40	0.57	0.81	1.82	0.85	0.36
**Number of metastatic sites**																
1, 2	16	2.07	1.17		2.54	0.92		3.09	2.40		1.44	0.62		1.39	0.71	
3, 4	14	1.83	0.86	0.53	2.54	2.36	0.99	2.77	1.61	0.67	1.40	0.52	0.84	2.01	1.00	0.06
**Prior therapy for MBC**																
No prior therapy	5	2.44	1.26		2.91	1.32		2.20	1.52		1.76	0.61		1.64	1.04	
Endocrine therapy	14	1.90	0.82	0.29	2.92	2.20	0.99	2.90	1.08	0.28	1.44	0.47	0.24	1.72	1.03	0.88
Cytotoxic chemotherapy	13	1.88	1.05	0.35	2.10	0.98	0.16	3.14	2.86	0.50	1.20	0.56	0.08	1.61	0.91	0.95
Anti-HER2 therapy	7	1.44	0.75	0.12	1.76	1.01	0.06	4.07	3.67	0.29	1.08	0.52	0.03	1.58	0.78	0.91

### Correlation between *PD*-*L1* and *FOXP3* expression in PBMCs and IFN-γ and TGF-β serum levels

**Fig. 3:**
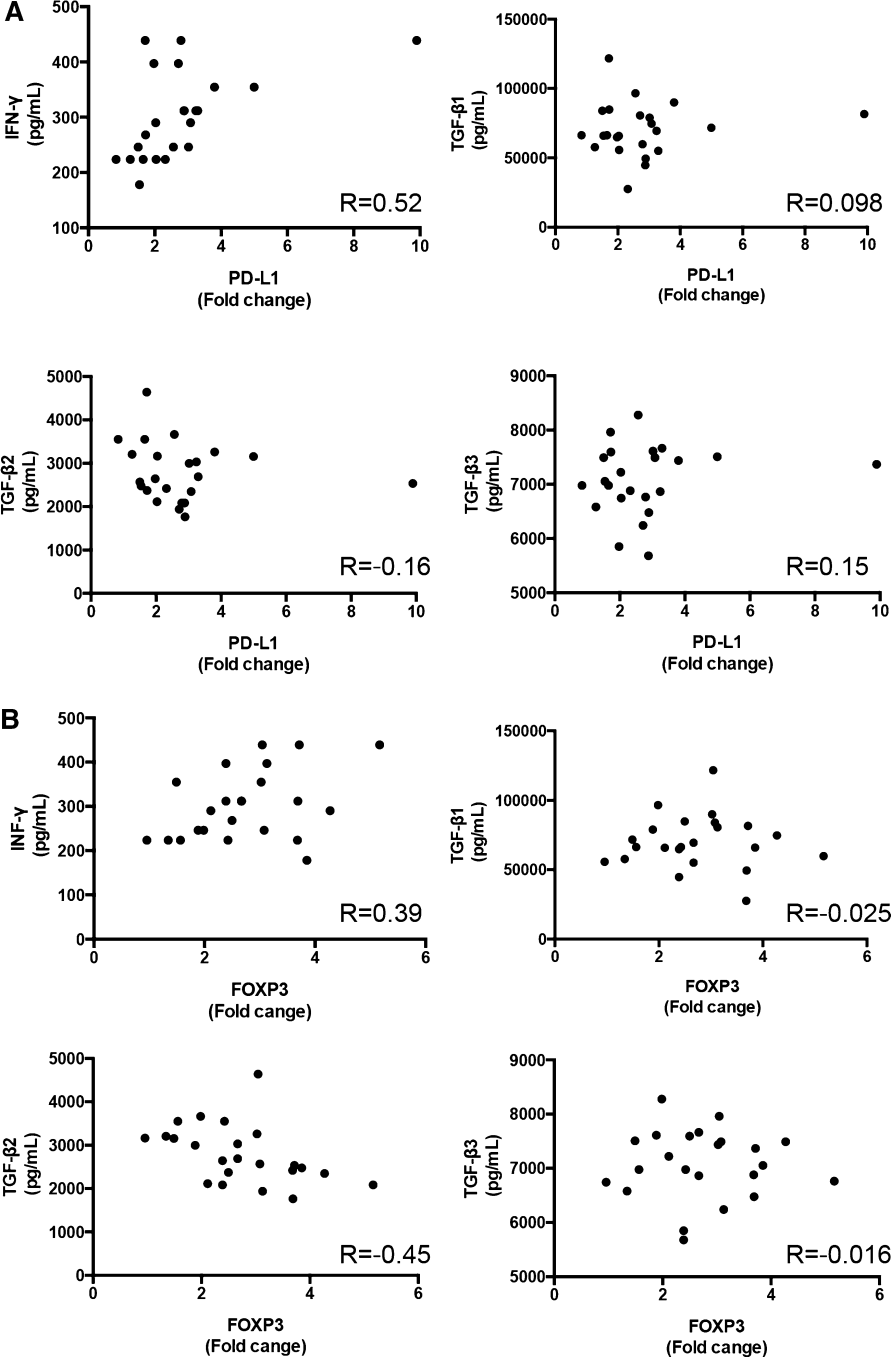
Correlation between *PD*-*L1* and *FOXP3* expression in PBMCs and interferon (IFN)-γ and transforming growth factor (TGF)-β levels in serum. **a** Correlation between *PD*-*L1* in PBMCs and IFN-γ and TGF-β1–3 in serum. **b** Correlation between *FOXP3* in PBMCs and IFN-γ and TGF-β1–3 in serum. *R* coefficient correlation value

We checked the correlation between *PD*-*L1* expression in PBMCs and cytokine levels in the serum of MBC patients. *PD*-*L1* expression correlated with IFN-γ (*R* = 0.52, *p* = 0.01), but did not correlate with TGF-β1 (*R* = 0.098, *p* = 0.66), TGF-β2 (*R* = −0.16, *p* = 0.48), or TGF-β3 (*R* = 0.15, *p* = 0.51) (Fig. 3a). Conversely, *FOXP3* expression correlated with TGF-β2 (*R* = −0.45, *p* = 0.03), but did not correlate with IFN-γ (*R* = 0.39, *p* = 0.067), TGF-β1 (*R* = −0.025, *p* = 0.91), or TGF-β3 (*R* = −0.016, *p* = 0.94) (Fig. 3b).

## Discussion

**Fig. 4:**
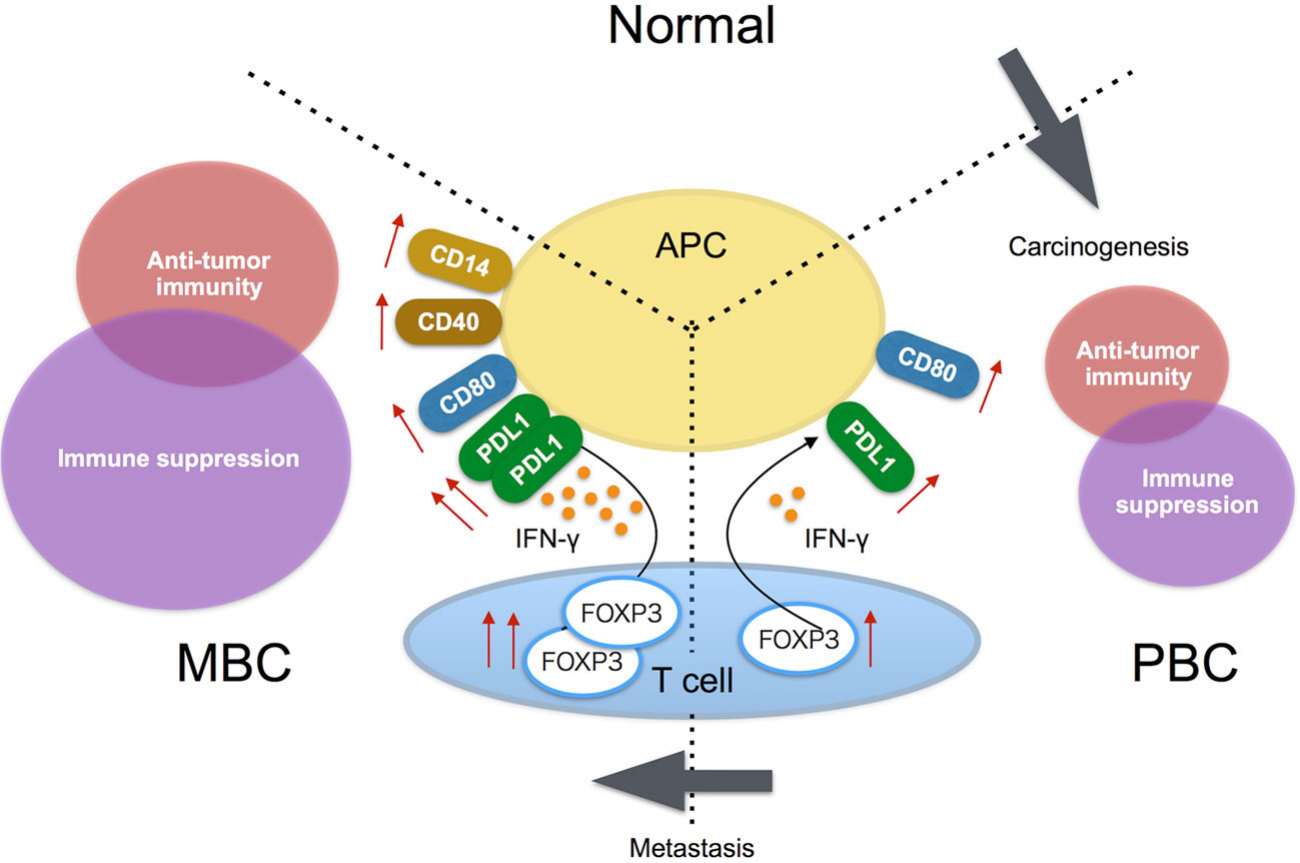
Graphical abstract of this study. *APC* antigen-presenting cells, *PBC* primary breast cancer, *MBC* metastatic breast cancer

Recently, biomarker analysis of immune cells was reported in the cancer microenvironment: TILs by immunohistochemistry, tumor digestion, and gene expression analysis of formalin-fixed and paraffin-embedded tissue samples, and flow cytometry analysis of immune checkpoint-related protein expression including inhibitory and activation molecules [13–16]. Additionally, PD-L1 expression by TILs rather than tumor cells was shown to be more predictive of the response to PD-1 pathway blockade [11, 12]. Thus, biomarker analysis has become increasingly importantly especially in immunotherapy to cancer. In this study, we prospectively validated PBMCs by qRT-PCR because PBMCs can be collected in a less invasive manner than tumor biopsy and can be compared quantitatively to other types of immune analysis. In fact, we show here that up-regulation of the mRNA expression levels of *PD*-*L1*, *FOXP3*, *CD80*, *CD40*, and *CD14* in PBMCs is associated with breast cancer. In addition, we found that the expression of these genes in PBMCs could be used to define three types of up-regulation: *CD80* was BC-specific, *CD40* and *CD14* were MBC-specific, and *PD*-*L1* and *FOXP3* were linear (Fig. 4).

The development of human cancer is a multistep process characterized by the accumulation of genetic and epigenetic alterations that drive or reflect tumor progression. These changes distinguish cancer cells from their normal counterparts, allowing tumors to be recognized as foreign by the immune system [17–19]. However, tumors are rarely rejected spontaneously, reflecting their ability to maintain an immunosuppressive microenvironment [20]. Immune checkpoint pathways strongly down-regulate T cell activation with the intent of keeping nascent T cell responses in check and reducing the likelihood of an immune attack against normal tissues. The myriad of genetic and epigenetic alterations that are characteristic of all cancers provide a diverse set of antigens that the immune system can use to distinguish tumor cells from their normal counterparts. In the case of T cells, the ultimate amplitude and quality of the response, which is initiated through antigen recognition by the T cell receptor, is regulated by a balance between co-stimulatory and inhibitory signals [21].

CD40, a tumor necrosis factor receptor superfamily member, is primarily expressed on antigen-presenting cells (APCs), such as dendritic cells and monocytes [22]. A recent report showed that anti-CD40 treatment induces PD-L1 up-regulation on tumor-infiltrating monocytes and macrophages, which was strictly dependent on T cells and IFN-γ [23]. Our data showed the up-regulation of *CD40*, *CD14*, and *PD*-*L1* in MBC (Fig. 2). These findings suggest that APCs are activated by neoantigens from a metastatic breast tumor and their activation leads to the activation of the checkpoint pathway, such as the up-regulation of *PD*-*L1* expression. In fact, *PD*-*L1* expression was correlated with IFN-γ levels in MBC (Fig. 3); thus, our gene expression analysis of PBMCs might reflect immune elimination and immune escape in the breast cancer microenvironment of the peripheral blood.

CD80 was found on activated APCs that provide a co-stimulatory signal necessary for T cell activation and survival. It is the ligand for two different proteins on the T cell surface: CD28 and CTLA4 [24]. CTLA4 interacts with both CD80 and CD86 with higher affinity and avidity than does CD28, with the CTLA4-CD80 interaction being the strongest and the CD28-CD86 interaction being the weakest [25]. Therefore, up-regulation of CD80 may indicate T cell tolerance in the breast tumor microenvironment, CD80 has the potential to be used as a CD80/CD86–CTLA4 pathway blocking therapy. However, in autoimmune diseases, the superior affinity of CTLA4 for its ligands led to the use of a CTLA4-immunoglobulin fusion protein (CTLA4-Ig) as an inhibitor of immune responses in vivo; the rationale being that it would bind to CD80 and CD86 and block their interaction with CD28 [26, 27]. Further investigation is required in vivo and in translational research of CD80 function in the cancer microenvironment.

The innate resistance of tumor cells to T cells is caused by activation of the AKT pathway, which leads to the up-regulation of PD-L1 expression on tumor cells [28]. On another front, adaptive resistance is generated by IFN-γ-induced PD-L1 expression on either tumor cells themselves or on immune cells (macrophages, myeloid suppressor cells, dendritic cells, or even lymphocytes) in the tumor microenvironment. Previous reports suggested that the up-regulation of PD-L1 and regulatory T cells in the cancer microenvironment in vivo depended on IFN-γ levels [23, 29]. Our data also showed that *PD*-*L1* and *FOXP3* were up-regulated in PBMCs in a linear pattern and correlated with IFN-γ serum levels (Figs. 2d, 3). These data indicate that up-regulation of *PD*-*L1* and *FOXP3* in PBMCs may be a result of adaptive resistance to T cells and their expression is regulated by IFN-γ levels in the breast cancer microenvironment.

This is the first report to demonstrate that the expression of immune genes in PBMCs is associated with breast tumor burden. In addition, one of the strong points of our research is that our samples were very high quality, as mRNA was extracted from PBMCs within 1 h from blood collection, and then preserved at a low temperature. Almost all previous studies preserved PBMCs before total RNA was extracted, regardless of the fact that the characteristics of PBMCs can be changed easily in the time from blood collection to freezing [6].

Our study is limited in that we performed gene expression analysis in PBMCs and not in a specific immune cell subset. However, from the clinical point of view, the study of PBMCs rather than the isolation of immune cell subsets requires less technical complexity and less sample processing time; although from an immunological point of view, gene expression analysis of specific immune cell subsets is more interesting and important. Another limitation of the current study is that we focused our attention on 16 major immune regulatory genes because they are associated with the immune checkpoint pathway and are therefore candidates for inhibiting T cell function at the tumor site. Several other candidate inhibitory mechanisms have been described, including the secretion of cytokines, such as TGF-β, interleukin (IL-4), and IL-10. Although we have not found these to be associated with *PD*-*L1* or *FOXP3* expression in PBMCs in breast cancer metastases, these factors could nonetheless contribute to immune evasion. This study is a plot setting to evaluate mRNA expression in PBMCs in breast cancer patients and sample size calculation was performed without various factors that may influence immune status such as cancer phenotype, therapeutic status and metastatic status. To overcome these limitations, we have started analysis of PBMCs in breast cancer by RNA sequencing as our next research project.

In conclusion, our data showed that the mRNA expression levels of *PD*-*L1*, *FOXP3*, *CD80*, *CD40*, and *CD14* in PBMCs were associated with breast cancer burden. Understanding the roles of these various interactions in breast cancer may become highly relevant for the development of immunomodulatory drugs and the discovery of biomarkers predictive of therapeutic response.
